# Media Agenda Setting Regarding Gun Violence before and after a Mass Shooting

**DOI:** 10.3389/fpubh.2016.00291

**Published:** 2017-01-09

**Authors:** Jared Michael Jashinsky, Brianna Magnusson, Carl Hanson, Michael Barnes

**Affiliations:** ^1^Department of Health Promotion and Behavior, University of Georgia, Athens, GA, USA; ^2^Department of Health Science, Brigham Young University, Provo, UT, USA

**Keywords:** gun violence, media agenda setting, mass media, violence prevention, news media

## Abstract

Gun violence is related to substantial morbidity and mortality with surrounding discussions framed and shaped by the media. This study’s objective was to explore national news media’s reporting of gun violence around a mass shooting. National news pieces were coded according to categories of gun violence, media frames, entities held responsible, responses, and reporting of the public heath approach. Individuals were held responsible for gun violence in 63% of pieces before and 32% after the shooting. Lawmakers were held responsible in 30% of pieces before and 66% after. Background checks were a proposed gun violence prevention method in 18% of pieces before and 55% after Sandy Hook, and lethality reduction of firearms was in 9% before and 57% after. Following a mass shooting, the media tended to hold government, not individuals, primarily responsible. The media often misrepresented the real picture of gun violence and key public health roles.

## Introduction

In the 4 years, since the Sandy Hook Elementary School mass shooting, where 27 were killed, over 18 other prominent shootings have since occurred in the US (1). A total of 32,888 individuals lost their lives due to firearms in 2013 with an additional 84,258 individuals experiencing non-fatal firearm related injuries (2, 3). Possessing a firearm is associated with an increased risk of being murdered or assaulted with a firearm as opposed to offering protection (4, 5). Firearms also play a significant role in suicide risk as a highly lethal means of suicide when available to many individuals who experience passing or impulsive suicidal thoughts (6). The social costs of gun violence in the US were $174 billion in 2010, $5.1 million per fatality, and $645 per gun (7).

The gun debate often results in political and societal gridlock and has not resulted in public policy that either major political party finds totally acceptable (8). Proponents of firearm safety legislation argue that laws to expand background checks, reduce firearm lethality, and many other approaches are sensible and will prevent death and injury; however, proponents of more open firearm policies argue that having guns in the hands of good people will promote safety and that restricting access to firearms will not prevent gun violence in the US. These opposing viewpoints, the effects of special interest groups, the second amendment, and steady firearm fatalities have mixed to create intense partisanship when dealing with gun violence in the US.

Clearly, the media plays important roles in the gun violence discussions by how they discuss and describe the issues. Through the process of framing, the media defines the problem, diagnoses the cause, and offers or justifies solutions for the problem (9). This type of media effect primarily characterizes *how* news reports are understood by audiences (10), which may change the way audiences view the problem and attribute responsibility. Media agenda setting is a related but unique media effect that sets an agenda for public discussion by the deliberate placement or amount of coverage given to topics or events with the goal of influencing public opinion and public policy (10, 11). Though both media effects have importance, this paper focuses primarily on agenda setting. Whereas the news media does not simply reflect reality but filters and shapes it (12), they become a gatekeeper to control “what” the public sees by devoting more or less of its online or print news pieces, screen time, or radio space to specific issues. The populous in turn places a greater or lesser interest in issues depending on how much attention is given to it by the news media. Evidence supports these premises: “people learn from the emphasis of issues in the news media first and foremost which issues are important [in addition to those that directly] affect their life” (13). Agenda setting has been used by researchers in diverse areas such as foreign policy, the US budget deficit, and the government response to Hurricane Katrina (11, 14, 15).

The public health approach has been increasingly applied to a variety of social issues in the world and involves four steps: define and monitor the problem, identify risk and protective factors, develop and test prevention strategies, and assure the widespread adoption of proven strategies including education, services, and public policy (16). This approach has effectively addressed important health issues including alcohol abuse, tobacco, and motor vehicle accidents (17, 18). Since policy-makers are likely to be effected by media, how well the media focuses on the public health approach when discussing gun violence could influence how well the public and lawmakers respond to gun violence in the US. To the authors’ knowledge, no study has looked at agenda setting relating to gun policy issues in the media surrounding Sandy Hook or the public health approach. The last agenda setting study relating to gun policy was conducted by Schnell who analyzed the media and political group’s discussion of the Brady Bill and Assault Weapons Ban (19), from which the present study is based.

The purpose of the study was to explore what agenda prominent national newspapers in the US are setting when discussing gun violence in the US before and after the Sandy Hook mass shooting. Specifically, the following hypotheses were tested:

H1: The media will hold government entities more responsible for gun violence prevention after the Sandy Hook mass shooting.
H2: The media will increase the frequency and/or elevate the prominence of stories reporting gun violence after the Sandy Hook mass shooting.
H3: The media will not frequently report aspects of the public health approach when discussing gun violence before or after Sandy Hook mass shooting.

## Materials and Methods

### Article Selection

A total of 2,270 English language US news pieces published between September 14, 2012 and April 25, 2013 were selected from three new outlets [The New York Times (NYT), The Wall Street Journal (WSJ) and Washington Post] using Factiva. These national papers were selected as they have a large readership (20, 21), play a lead role in setting the media agenda for subnational newspapers, have a strong track record as agenda setters, and have a history in agenda setting literature (22–26). Only news pieces with a minimum of 400 words (27) and containing at least three instances of the words “firearm” or “gun” with at least one occurring in the first 200 words of the article were included. Blogs, transcripts, abstracts, and duplicate articles were excluded.

The sampling frame was separated into four different strata by type (stories vs. editorials) and publication date (September 14–December 14, 2012 vs. December 15, 2012 to April 25, 2013). The frame included 154 stories and 12 editorials published on or before December 14, 2012 and 1,985 stories and 165 editorials published after December 14, 2012. All news pieces on and prior to December 14, 2012, the editorials after December 14th, and a random sample of 255 stories published after December 14th were included in the study sample, resulting in 586 news pieces for coding. The random sample of only the news stories published after December 14, 2012 ensured that enough news pieces were coded in the other three categories to ensure reliable representations were recorded and reduced the number of news stories to a manageable number. Taking a simple random sample across all categories would have resulted in too few news pieces in the other three categories. Though this reduced the quantity of after Sandy Hook news pieces coded, the random nature of the sample allowed for the estimated values from the sample to still reflect the populations before and after Sandy Hook news pieces.

Of the 586 news pieces, a total of 375 were found to meet the inclusion criteria during coding. Two research staff coded the selected news pieces. A 10% overlap in coding was completed and percent agreement and Kappa scores calculated to examine inter-rater reliability.

### Measures

News pieces were coded for prominent agenda setting and framing perspectives including categories of gun violence discussed, policy and media frames, entities held responsible for gun violence, proposed methods for reducing gun violence, and reporting of the public health approach.

#### Categories

Mass homicide was defined using the FBI’s definition of four murders in a single incident (28).

#### Framing

Policy and media frames from Callaghan and Schnell’s (19) were selected for the present study if they were found often in their review of media framing during a gun control debate in 2001 or if the frame appeared salient to the 2012 debate. Frames coded included culture of violence, political contests, feel-good laws, sensible legislation, special interests, will of the people (pro-gun control), will of the people (anti-gun control), constitutional rights, court challenge, states’ rights, constitutional limits, public safety, gun’s don’t kill people, people do, and guns deter crime. As with previous research on framing (11, 29–32), news pieces were also coded for whether the frame was thematic, episodic, or a combination approach. Thematic approaches generally covered gun violence in broad terms on a societal level while episodic approaches focused on telling the story of perpetrators or victims of single occurrences of gun violence in a story-telling fashion.

#### Responsibility

Entities held responsible for the gun violence problem included individuals, families, schools, police, justice system, entertainment media, gun manufacturers, gun distributors, lawmakers, religious institutions, and executive government.

#### Prevention Methods

Gun violence prevention methods included background checks, increasing severity of punishment, safety engineering, safe storage laws, improve emergency medical response, lethality reduction, metal detectors in schools, increased mental health services, legislation to reduce gun trafficking, youth mentoring, and better enforcement of existing laws. Prevention methods were selected by investigator consensus to represent a broad range of approaches as well as the most common approaches discussed.

#### Public Health Approach

Finally, news pieces were coded on a “yes” or “no” basis for the following three questions aimed at assessing how well the media reported on using the public health approach to curb gun violence as described by the Centers for Disease Control and Prevention (CDC): Does the article cite the importance of research, generating data, risk factors, etc.? Does the article mention using “evidenced based” practices? and Does the article mention evaluating the implementation of the methods used to prevent gun violence? (16).

### Statistical Analysis

Frequencies and proportions described the distribution of agenda setting themes overall and before and after Sandy Hook. Chi-square tests were used to test for differences in proportion of news pieces before and after Sandy Hook that discussed specific agenda setting domains. To examine the differences between stories only before and after Sandy Hook, a sensitivity analysis was conducted excluding all editorial pieces. All analyses were conducted using SAS 9.4 (SAS Institute Inc., Cary, NC, USA).

## Results

A total of 375 eligible news pieces were identified of which 89 (24%) were published prior to the Sandy Hook shooting. A portion of the variables’ inter-rater reliabilities failed to reach acceptable standards (Kappa > 0.4) and were removed from the results. The minimum Kappa value of 0.4 was based on Landis and Koch interpreting a Kappa between 0.41 and 0.6 as moderate inter-rater reliability (33).

The majority (54%) of news pieces were published in the Washington Post with 32% appearing in the NYT and 14% in the WSJ. The distribution of news pieces before and after Sandy Hook is similar by publication source [χ^2^(2, *N* = 375) = 2.69, *p* = 0.26, Φ_Cramer_ = 0.09]. The mean word count was 922.80 (SD = 867.00) words for those published prior to Sandy Hook and 900.70 (SD = 509.10) words for those published after [*t*(107.51) = 0.23, *p* = 0.82, *d* = 0.04]. The most common type of gun violence discussed was mass homicide (59%), followed by homicide (32%). Suicide by firearm (10%) and gun accidents (4%) were less common themes.

### Policy and Media Frames

The policy and media frames most common among the reliable estimates were special interests (37%) and sensible legislation (16%) (see Table 1). All three frames were significantly more cited following the Sandy Hook shooting as compared to before including will of the people (pro-gun control), special interests, and sensible legislation.

**Table 1:** **Proportion of news pieces with framing, entities held responsible, and methods for reducing gun violence by publication timing, US, 2013**.

	Before Sandy Hook (*n* = 89)	After Sandy Hook (*n* = 286)	Total (*n* = 375)
**Policy and media frames**			
Sensible legislation	5.62	19.58**	16.27
Special interests	24.72	40.21**	36.53
Will of the people (pro-gun control)	4.49	13.64**	11.47
**Entities held responsible**			
Individuals	62.92	31.82**	39.20
Families	8.99	6.99	7.47
Schools	6.74	10.14	9.33
Lawmakers	30.34	66.43**	57.87
Religious institutions	1.12	1.75	1.60
Executive government	35.96	44.41	42.40
**Methods for reducing gun violence**			
Background checks	19.10	55.24**	46.67
Safety engineering	0.00	1.05	0.80
Safe storage laws	1.12	2.10	1.87
Lethality reduction	8.99	56.99**	45.60
Metal detectors in schools	0.00	5.24	4.00
Increased mental health services	2.25	15.73**	12.53
Legislation to reduce gun trafficking	6.74	15.73*	13.60
Youth mentoring	1.12	0.35	0.53

*p < 0.05.

**p < 0.01.

### Entities Held Responsible

Across all news pieces the most common persons or entities held responsible for gun violence issues were lawmakers (58%), the executive government (42%), and individuals (39%) (see Table 1). These remained the top three entities both before and after Sandy Hook, however, the rankings change. Prior to Sandy Hook, 63% of news pieces sampled mentioned holding individuals responsible for gun violence, which reduced to 32% following Sandy Hook [χ^2^(1, *N* = 375) = 27.55, *p* < 0.001, Φ = 0.27]. Conversely, lawmakers were held responsible for gun violence in just 30% of news pieces before the shooting and 66% of news pieces following the shooting [χ^2^(1, *N* = 375) = 36.27, *p* < 0.001, Φ = −0.31].

### Methods for Reducing Gun Violence

Across all news pieces, background checks (47%), lethality reduction (46%), legislation to reduce gun trafficking (14%), and increased mental health services (13%) were the most commonly mentioned methods for reducing gun violence (see Table 1). The most commonly mentioned methods before Sandy Hook for reducing gun violence were background checks (19%), lethality reduction (9%), and legislation to reduce gun trafficking (7%). Following Sandy Hook, the most commonly mentioned methods were lethality reduction (57%), background checks (55%), legislation to reduce gun trafficking (16%), and increased mental health services (16%). Comparing before and after Sandy Hook news pieces, there were significant increases in the number of news pieces that discussed background checks [χ^2^(1, *N* = 375) = 35.63, *p* < 0.001, Φ = −0.31], lethality reduction [χ^2^(1, *N* = 375) = 63.05, *p* < 0.001, Φ = −0.41], increased mental health services [χ^2^(1, *N* = 375) = 11.26, *p* < 0.001, Φ = −0.17], and legislation to reduce gun trafficking [χ^2^(1, *N* = 375) = 4.67, *p* = 0.03, Φ = −0.11]. Two methods appeared in no news pieces prior to Sandy Hook, but did appear after. Although the difference is not testable with a chi-square test, safety engineering was mentioned in 3 pieces and metal detectors in 15 pieces following Sandy Hook.

### Public Health Approach

None of the public health approaches were sufficiently reliable to report; however, only the importance of research was noted in over 10% of the sample. Even the importance of research at its peak was only in 17% of the after Sandy Hook sample. Even though the measures were unreliable, it appeared likely that the public health approaches were quite rare or at least not widely reported.

### Editorials vs. Stories

Roughly, half (*n* = 145) of news pieces post-Sandy Hook were editorials. To determine the differences between editorials and articles, we conducted a stratified analysis of the post-Sandy Hook news pieces. Generally, editorials and stories mentioned the same constructs; however, there was one notable difference. A higher percentage of editorials compared to stories mentioned sensible legislation [28 vs. 11%; χ^2^(1, *N* = 375) = 14.12, *p* < 0.001, Φ = −0.22].

### Sensitivity Analysis of Articles Only

Given the differences between stories and editorials and the high proportion of editorials among post-Sandy Hook news pieces, we conducted a sensitivity analysis of the stories only to examine differences before and after Sandy Hook. The results were qualitatively similar to the results comparing all news pieces with one addition, post-Sandy Hook stories were more likely to hold the executive government responsible [50 vs. 30%; χ^2^(1, *N* = 223) = 7.78, *p* = 0.005, Φ = −0.19] (data not shown).

## Discussion

The purpose of the study was to explore the agenda setting function of prominent national newspapers in the US regarding gun violence in the US before and after the Sandy Hook mass shooting and how well the issue was framed in terms of the public health approach. Comparing the results of this study to national death rates, 63% of US gun violence deaths occur from suicides while a little over 10% of the news stories discussed suicide. Additionally, 33% of US gun violence deaths occur from homicides while over 90% (59% mass homicide, 32% homicide) of the news stories discussed the topic. These differentials are not uncommon, as researchers in other areas have demonstrated inaccurate risk perceptions (34). Clearly, the media tends to emphasize news stories to reflect its needs or purposes. While mass shootings are particularly devastating, the US may be inaccurately placing a majority of its media, policy, and national discussions on a relatively small component of gun violence. More media focus on firearm suicides and non-mass shooting homicides could make a larger impact on firearm related morbidity and mortality in the US.

On January 16, 2013, President Obama called on the Federal Government to respond to recent massacres by promoting universal background checks for gun sales, the reinstatement and strengthening of the assault weapons ban, limiting ammunition magazines to a 10-round capacity, providing schools with resource officers and counselors, putting more police officers on the streets, establishing stronger punishments for gun trafficking, and offering more comprehensive insurance coverage for mental health. These calls from the executive government to address gun violence after Sandy Hook mirrored some of the changes in the media landscape. For example, prior to Sandy Hook, news media attributed most of the responsibility for gun violence to individuals, lawmakers, and then executive government. Following Sandy Hook, however, the news media attributed most of the responsibility for gun violence to lawmakers, executive government, and then individuals. A significant increase in the percentage of news pieces holding lawmakers responsible after Sandy Hook supported the hypothesis that the media would hold government entities more responsible for gun violence prevention after the Sandy Hook mass shooting; however, the increase in the executive government being held responsible was not significantly different after Sandy Hook.

Additionally, the media framed gun violence reporting differently after Sandy Hook by increased use of the sensible legislation, special interests, and will of the people (pro-gun control) frames, supporting the hypothesis that media framing would change after the Sandy Hook mass shooting. However, stories about gun accidents and suicides dropped noticeably after Sandy Hook in favor of homicide stories that emphasized the need for various societal responses. The media emphasized reduction methods noticeably different after Sandy Hook. For example, lethality reduction measures (five times greater), background checks, and legislation to reduce gun trafficking were noted (two times greater). Such shifts not only shape discussion and thought, but also tend to influence the perceptions, norms and values of many—particularly decision makers (35).

To expand on the CDC’s public health approach to gun violence, Hemenway and Miller discuss five additional components: the approach focuses on populations over individuals, aims to prevent the problem as upstream as possible, uses a systems approach attempting to make mistakes difficult and less harmful when they occur, examines all potential approaches and engages many segments of the population in the solution, and lastly focuses on shared responsibilities over attributing blame to certain individuals (36). The upstream public health approach has proven successful around the world (37–40) and supports public health’s interest in health-in-all-policies (41).

In terms of focusing on populations over individuals, the sampled news pieces largely ignored many of the entities that could have been responsible for the problem, supporting the hypothesis that the media would not frequently report aspects of the public health approach when discussing gun violence. For example, only individuals, legislators, and the executive government received significant discussion. For the news media to effectively support discussion of the public health approach, more should be done to diversify the discussion of entities responsible for both the problem and necessary changes. Instead of simply viewing gun violence as a problem that the president needs to fix, or that the perpetrators need to stop, a broader discussion would highlight complimentary approaches and partners who can help solve the problem. Media emphasis could be placed on decreasing the saturation by treating these acts like more ordinary crimes could make them less ordinary, or telling a different story like describing potential perpetrators or would-be suicides who turned for help from others before acting. The thought behind such actions, similar to an overriding public interest to protect the privacy of sexual assault victims, the reputation of the accused, and public safety (avoidance to publish the making of a bomb), there is a need for public health to help the media focus on the most important issues (42).

In terms of examining all potential approaches, the news pieces did not reflect the full variety of potential approaches for reducing gun violence, again supporting the hypothesis that the media would not frequently report aspects of the public health approach when discussing gun violence. Background checks and lethality reduction of firearm technology were the only two approaches that received significant discussion. Lastly, in terms of using a system approach to make mistakes difficult, approaching gun violence through safety engineering of the firearm technology was only discussed in less than 1% of the news pieces. Technologies such as magazine safeties, personalized firearms, loaded chamber indicators, and grip safeties exist that can make guns safer (43); however, discussion of these approaches was virtually absent in the news media reviewed. While more downstream in terms of prevention, the news pieces in this study frequently discussed lethality reduction as an approach to making gun violence less harmful when it occurs.

Public health should emphasize the value of prevention in all gun violence issues. Further focusing the media’s attention on upstream prevention approaches would help align the media’s agenda and public opinion on the most effective approaches available. Effective reporting in the media could steer the gun violence discussion from a philosophical debate toward the more scientific and successful public health approach.

### Limitations

While this study contributes to the understanding and discussion of the media’s reporting of gun violence in the US, it is important to interpret the results with the following limitations in mind. First, Factiva improperly coded some news pieces resulting in their inclusion in the study’s random sample. This led to a reduced sample size after they were excluded for conflicting with preset inclusion and exclusion criteria. Second, inter-rater reliabilities of some subscales were lower than desired. Though the Kappa > 0.4 standard was not as strict as some used in news media studies, a lower acceptable reliability needed to be balanced against the dearth of gun violence research in the US. Of the unreliable variables that were not reported in the results, only political contests, constitutional rights, thematic vs. episodic, and police were noted in over 10% of the sample. Even though the unreported measures were unreliable, it appeared likely that most did not occur frequently. Future work should conduct more pre-testing of their coding systems to ensure higher inter-rater reliability levels for all study variables. Third, the descriptive nature of the study primarily prompts questions and discussion of the issues at hand. The study falls short of demonstrating causal pathways that could be manipulated to improve the media’s reporting or gun violence in the US.

## Conclusion

Following a mass shooting, the media tended to hold government, not individuals, primarily responsible. However, the media often misrepresented the real picture of gun violence by largely focusing on mass shootings and did not discuss key public health approaches for preventing gun violence. Public health professionals should strive to promote effective policies aligning with the public health approach that address all types of gun violence. Efforts to promote better media coverage could improve the US response to gun violence.

## Author Contributions

JJ contributed to the paper by designing the study, coding news pieces, and writing the paper. BM contributed to the paper by conducting analyses and writing the paper. CH contributed to the paper by designing the study and writing the paper. MB contributed to the paper by designing the study and writing the paper. All authors approve of the submitted manuscript and are accountable for the paper.

## Conflict of Interest Statement

The authors declare that the research was conducted in the absence of any commercial or financial relationships that could be construed as a potential conflict of interest.
